# The Absorption, Distribution, and Excretion of 18 Elements of Tibetan Medicine Qishiwei Zhenzhu Pills in Rats with Cerebral Ischemia

**DOI:** 10.1155/2021/4508533

**Published:** 2021-12-28

**Authors:** Yinglian Song, Ke Fu, Dewei Zhang, Min Xu, Ruixia Wu, Xueqing Xiong, Xianwu Liu, Lei Wu, Ya Guo, You Zhou, Xiaoli Li, Zhang Wang

**Affiliations:** ^1^College of Pharmacy, Chengdu University of Traditional Chinese Medicine, Chengdu 611137, China; ^2^Wanzhou Institute for Drug and Food Control, Chongqing 404000, China; ^3^College of Ethnomedicine, Chengdu University of Traditional Chinese Medicine, Chengdu, 611137, China

## Abstract

The aim of this study is to determine 18 elements in Tibetan medicine Qishiwei Zhenzhu pills (QSW) and their absorption, distribution, and excretion in rats with cerebral ischemia. Microwave digestion and inductively coupled plasma mass spectrometry (ICP-MS) were used to determine 18 elements of QSW in simulated gastrointestinal (GI) juice. Rats were given QSW (66.68 mg/kg) followed by middle cerebral artery occlusion (MCAO). Sham rats received saline and were not subjected to MCAO. ICP-MS was applied to determine the content of 18 elements in hepatic venous blood, abdominal aortic blood, brain, liver, kidney, hair, urine, and feces 24 h after MCAO. In vitro results showed that the extraction rate of Mn, Cu, Sr, Pb, Au, and Hg of QSW in gastric juice (1 h) was higher than that in water, and the contents of Cu, Au, Sr, and As were higher in intestinal juice (4 h) than in water. In vivo results showed that the contents of elements in the blood were quite low, and QSW increased Ni, Cr, Sr, Co, and V in artery blood and decreased V in venous blood. Elements in the tissues were also low, and QSW increased brain Li but decreased Cr and Cd; QSW increased kidney Ag and Cs and liver Mn but decreased liver Ni. QSW increased urinary excretion of Li, Sr, Hg, Cs, and V; QSW increased Hg content in hair but decreased Ni. Stool is the main excretion pathway of the elements in QSW, with Ba, Mn, Sr, Cd, V, Cu, Cs, Li, Pb, Ag, Hg, Cr, As, and Co the highest. In summary, this study examined the distribution of 18 elements in QSW-treated MCAO rats. The accumulation of these elements in blood and tissues was extremely low, and the majority was excreted in feces within 24 h, highlighting the importance of the gut-microbiota-brain axis in QSW-mediated brain protection.

## 1. Introduction

Cerebral ischemia is the commonest type of cerebral infarction [1]. On the basis of vascular wall lesions caused by cerebral atherosclerosis, vascular stenosis, occlusion, or thrombosis may result in ischemia and hypoxic necrosis of local brain tissues due to interruption of the blood supply, which result in corresponding symptoms and signs in the nervous system [2]. Cerebral ischemia is equivalent to ischemic stroke in traditional Chinese medicine, “Baimai” disease in Tibetan medicine, and “Sa” disease in Mongolian medicine. The usual symptoms of “Baimai” disease are skewed mouth and eyes, numbness in limbs, rigidity, angular arch reflex, paralysis, hemiplegia, unconsciousness, and head tremors [3].

Qishiwei Zhenzhu pills (QSW), of which the Tibetan name is, are also known as Ranna Sangpei and was first recorded in the great classical work of Tibetan medicine *Si Bu Yi Dian*. QSW have the functions of tranquilizing the mind, activating meridians and collaterals, and harmonizing qi and blood. They are mainly used to treat “Baimai” disease, “Longxue” disorder, stroke, paralysis, hemiplegia, epilepsy, cerebral hemorrhage, and other diseases [4]. According to the published literature, there should be 32 kinds of plant medicine, 8 kinds of animal medicine, more than 20 kinds of mineral and gem medicine, and a mineral mixture (Zuotai) in QSW. QSW are a confidential prescription, only the names of some medicines are disclosed, and the specific prescription proportion is unknown. Only 12 plant medicines, 8 animal medicines, 8 mineral and gemstone medicines, 4 metal medicines, and Zuotai are known and are listed in Supplementary Table 1 [5]. QSW consist of more than 10 metallic elements, such as gold, silver, and copper, more than 20 minerals and gemstones, such as sapphire and agate, and a mineral mixture Zuotai [6]. In particular, in accordance with the theories of Tibetan medicine, Zuotai is the main ingredient of many important Tibetan medicinal preparations. Zuotai is obtained from mercury and other minerals by a complex processing procedure, and hence QSW contain a large amount of mercury. The high mercury content in QSW results in serious health problems [7–9]. Therefore, the determination of the contents of mercury and other mineral components in QSW is very important for safety in clinical use.

The choice of detection method is important for the determination of trace elements, and a variety of different methods are used to detect elements. For example, Yang et al. [10] used synchrotron radiation X-ray fluorescence (SR-XRF) technology to quantify and semiquantify nine and fifteen elements in Tibetan herbal medicines and Tibetan medicine preparations, respectively. Flame atomic absorption (FAAS) with wet digestion method has also been used to quantitatively analyze various trace elements in Tibetan medicine Nan Hanshuishi [11]. In addition, the total mercury in the Tibetan medicine Dangzuo and the free mercury in the artificial gastric juice are also measured using the gold amalgam enrichment-atomic fluorescence (GAE-AFS) method [12]. Among a variety of detection methods, compared with traditional inorganic analysis technology, ICP-MS technology has the advantages such as lower detection limit, wider dynamic linear range, less interference, higher analysis precision, and analysis speed, and it can provide accurate isotope information and has a wide range of applications [13]. In medicine, it can be applied to the analysis of hair, blood samples, urine samples, biological tissues, biological mechanism research of proteins, enzymes, etc., drug quality control, etc. [14]. This research uses ICP-MS technology for experiment; the results will provide new ideas for the establishment of determination methods as well as the quality control and clinical rational application of QSW in the future [15].

As for the determination of trace elements in QSW, the following studies have been conducted: Suo et al. used inductively coupled plasma-atomic emission spectrometry (ICP-AES) to analyze and test more than 20 trace element components of QSW [16]. Li et al. used ICP-AES to determine the contents of 9 trace elements (Cu, Fe, Co, Mn, V, Zn, Mg, P, and K) in QSW and provided a theoretical basis for explaining their clinical effects on the basis of synergistic and antagonistic elements [17]. Li and Suo analyzed the long-term accumulation and toxic effects of trace elements present in QSW in animal tissues and organs. The results showed that most of the trace elements present in QSW did not accumulate in the heart, liver, kidneys, or other tissues and organs. Excessive amounts of heavy metals such as arsenic and cadmium were ingested after the administration of QSW and were mainly excreted through feces and urine [18]. Du et al. quantitatively analyzed the contents and distribution of toxic heavy metals (As, Hg, and Pb) in QSW on the basis of the findings of Li et al. [19]; but, at present, there have been no studies of the accumulation of trace elements in the body during the treatment of cerebral ischemia with QSW. In this study, we established a new, sensitive, selective, and accurate method for simultaneous quantitative analysis of the contents in the simulated GI juice and water of 18 elements in the QSW and their absorption, distribution, and excretion in rats with cerebral ischemia. By comparing the contents in the simulated GI juice and water of each element and their changes in the blood, distribution in the brain, liver, and kidney tissues, and excretion in hair, urine, and feces, the pharmacodynamic medicinal substance basis and toxicity of the QSW and its metabolism in the body can be found.

We have shown that QSW are effective in the treatment of cerebral ischemia injury [20]. The disposition of 18 elements during the treatment of MCAO by QSW could add our understanding of the protection mechanisms of QSW against cerebral ischemia injury. The results clearly demonstrated that 24 h after QSW administration to MCAO rats, these elements' accumulation in the blood and tissues were extremely low, and the amount found in feces were extremely high, implying these elements in the GI tract could modulate gut microbiota [21] to produce neuroprotection through the gut-microbiota-brain axis.

## 2. Materials and Methods

### 2.1. Drugs

QSW (batch number: 17103A, Guoyao Zhunzi: Z54020062) was purchased from Tibet Ganlu Tibetan Medicine Co., Ltd.

### 2.2. Reagents

Rh (internal standard stock solution [ISS]), In (ISS), Re (ISS), Bi (ISS), Li (standard solution [SS]), Be (SS), Sc (SS), V (SS), Cr (SS), Mn (SS), Co (SS), Ni (SS), Cu (SS), As (SS), Sr (SS), Ag (SS), Cd (SS), Cs (SS), Ba (SS), Pb (SS), Au (SS), and Hg (SS) all 22 reagents were produced by the National Nonferrous Metals and Electronic Materials Analysis and Testing Center. Pepsin and pancreatin were manufactured by Xinxiangsheng Biopharmaceutical Co., Ltd.

### 2.3. Instruments and Working Parameters

An inductively coupled plasma mass spectrometer (ICP-MS) (mode: ICAP-Q Series; Thermo Fisher Scientific, USA) was used with the following parameters: RF power 1.3 kW, carrier gas flow rate 1.14 L/min, sampling depth 6.8 mm, rectangular tube horizontal position 0.6 mm, auxiliary gas flow rate 0.9 L/min, spray chamber flow rate 0.98 L/min, and spray chamber pressure 2.85 × 105 Pa.

A microwave digestion apparatus (model: Milestone ETHOS A; Huashengda Instrument Equipment Co., Ltd.) was employed with the following parameters: (1) increase the temperature from room temperature to 120°C within 15 min and maintain for 5 min (1600 W); (2) increase the temperature to 150°C within 7 min and maintain for another 7 min (1600 W); and (3) within 7 min, increase the temperature to 190°C and maintain for 15 min (1600 W).

The following instruments were also used: an electronic analytical balance (model: XP204; Mettler-Toledo) and a constant temperature oscillator (model: SHZ-82; Guowang Instrument Manufacturing Co., Ltd.).

### 2.4. Preparation of Standard Solution

Gold working solutions with a mass concentration of 10 *μ*g/mL were formulated (The Hg element easily produces memory effect as well as adsorption effect.). Precision stock solutions containing 18 tested metal elements, including Li, Be, Sc, V, Cr, Mn, Co, Ni, Cu, As, Sr, Ag, Cd, Cs, Ba, Pb, Au, and Hg, were used at appropriate amounts and diluted clinically with 10% HNO_3_ to form mixed standard solutions with serial mass concentrations of each element: Li, Be, Sc, V, Co, Cd, Cs (0, 0.5, 2.0, 10, 25, 50 *μ*g/L); Cr, Mn, Ni, Cu, As, Sr, Ag, Ba, Au, Pb (0, 0.5, 2.0, 10, 25, 50) × 10^3^ *μ*g/L; Hg (0, 0.2, 0.5, 1.0, 1.5, 2.0) × 10^2^ *μ*g/L; the standard solutions of mercury were configured individually; and the other 17 elements were configured into mixed standard solutions. Precision stock solutions of internal standards containing Rh, Re, In, and Bi diluted with 10% HNO_3_ to a mixed internal standard solution at a concentration of 10 *μ*g/mL and used.

### 2.5. Preparation of Test Samples of QSW

The QSW were wrapped with filter paper and crushed (not with a mortar to prevent the introduction of heavy metals). Exactly 0.0500 g of the sample was poured slowly into a polytetrafluoroethylene (PTEF) digestion tank, which was soaked in HNO_3_ overnight, washed, and dried. Afterward, 10 mL of HNO_3_ and 2 mL of H_2_O_2_ (both of premium grade) were added, shaken slowly, fixed, placed under the microwave heating program, and digested for 60 min. After the digestion was completed, the microwave digestion apparatus was turned off and cooled for a while. The digestion tank was removed and cooled to room temperature. The test solution was transferred to a 50 mL volumetric flask and washed three to four times. A constant volume was achieved. Given that Hg is easy to be adsorbed, 0.1 mL of the sample was placed in a 50 mL volumetric flask and added with gold standard solution to stabilize for testing (equivalent to 500-fold dilution).

### 2.6. Sample Preparation of QSW after the Digestion of Simulated GI Juice

Artificial GI juice was prepared in accordance with the *Pharmacopoeia of the People's Republic of China* (2015 edition, Vol. IV) [22]. For the preparation of artificial gastric juice, 16.4 mL of diluted hydrochloric acid was added with 800 mL of water and 10 g of pepsin, shaken well, and diluted to 1000 mL with water. For the preparation of artificial intestinal juice, phosphate buffer (containing pancreatin) and 6–8 g of potassium dihydrogen phosphate were dissolved in 500 mL of water, and the pH was adjusted to 6.8 with 0.1 mol/L sodium hydroxide solution. In addition, 10 g of pancreatin was dissolved in an appropriate amount of water. The two liquids were then mixed, added with water, and diluted to 1000 mL.

Simulated GI juice digestion and water samples were prepared using the method of Jin's research group [23]: 0.5000 g of finely ground QSW (approximately equivalent to the amount taken by a patient with mild to moderate diseases) was placed in a 200 mL conical flask with 100 mL of artificial gastric juice. The solution was vortexed at 37°C for 1 h and filtered, and the residual liquid was washed three times with artificial gastric juice. The filtrate was diluted with water to 200 mL to obtain an extract of artificial gastric juice. Another portion of the same sample powder was placed in a 200 mL conical flask filled with 100 mL of artificial intestinal juice, vortexed at 37°C for 4 h, and filtered. The residue was washed three times with artificial intestinal juice, and the filtrate was diluted with water to 200 mL to obtain an extract of artificial intestinal juice. Another part of the same sample powder was placed in a 200 mL conical flask with 100 mL of water, and then two copies were made and vortexed at 37°C for 1 h and 4 h, respectively, filtered, and rinsed with clean water three times. The filtrate was diluted to 200 mL with water to obtain the water extract. Approximately 1 mL was obtained for each of these four extracts, placed into the PTEF digestion tank, and added 4 mL of HNO_3_ (premium grade). The remaining steps were similar to “Preparation of Test Samples of QSW” to prepare the test solution for testing.

### 2.7. Experimental Animals

Twelve male SD rats of SPF grade weighing 250–280 g were purchased from Chengdu Dashuo Experimental Animal Co., Ltd. (license number: SCXK [Sichuan] 2015-030, experimental animal quality certificate number: No. 51203500008744). The experiment was conducted in the National Medicine Resource Evaluation Laboratory of the Chengdu University of Traditional Chinese Medicine (the third-level scientific research laboratory of the State Administration of Traditional Chinese Medicine, NO.TCM-2009-320).

### 2.8. Animal Grouping and Test Drug Dosage

All male SD rats were divided into the following two large groups, each group with 6 animals: Sham operation group (abbreviated as Sham) and Qishiwei Zhenzhu pills group (abbreviated as QSW + MCAO) (66.68 mg/kg) [24, 25]. Based on the group, the rats were administered saline or QSW once through intragastric administration, and the administration volume was 10 mL/kg. The dosage of 66.68 mg/kg QSW is 4 times the daily dose for clinical adults, which is the optimal dose [26, 27].

### 2.9. Preparation of the MCAO Model

Performing animal modeling immediately after intragastric administration, refer to the predecessor model preparation method [26–29], separate the right common carotid artery (CCA), external carotid artery (ECA), and internal carotid artery (ICA) and ligate the ECA. Separate the pterygopalatine artery inward along the ICA and ligate at the bifurcation. Cut an incision between the distal and proximal ends of the ECA, insert the tie wire into the ICA about 20 mm, and suture the wound and sterilize it. The animal was kept warm until awoke after modeling. The middle cerebral artery was occluded for 2 hours and then reperfused. In the Sham group, only the wound was sutured and a model was not made.

### 2.10. Collection and Determination of Animal Samples

At 24 h after intragastric administration of saline in the Sham group and after successful modeling for 24 h in the QSW + MCAO group, 20% urethane solution was injected intraperitoneally (0.6 mL/100 g) to anesthetize the rats, and then the rats in each group were treated with heparin sodium anticoagulation tube to collect blood from the hepatic portal vein and abdominal aorta. After taking blood from the hepatic vein and abdominal aorta, the liver, brain, kidney, as well as feces, urine, and hair were quickly collected, and then the rats were killed with cervical dislocation. When taking urine, gently squeeze the abdomen of the rat's bladder to speed up the collection of urine. Abdominal aortic blood, hepatic portal vein blood, and urine samples were measured to 0.50 mL using a pipette. The feces were obtained from the colon, and the back hairs of rats were collected. The feces and hair samples were weighed at 0.5000 g. And the brain, liver, and kidney were taken, weighed 0.5000 g, and then were homogenized. Transfer each of the above samples into the PTEF digestion tank, and add 8 mL HNO_3_ (premium grade), same as “Preparation of Test Samples of QSW”; finally, transfer the digested test solution to a 25 mL volumetric flask to constant volume to be tested.

### 2.11. Statistical Analysis

SPSS 21.0 software was used for data analysis, and the data were expressed as mean ± SD. The independent sample *t*-test was performed for the comparison between two groups. When the test result is *p* < 0.05, the difference between groups is considered to be significant.

## 3. Results

### 3.1. In Vitro Quantitative Analysis of Minerals and Heavy Metals in QSW by ICP-MS

#### 3.1.1. Methodological Investigation

Referring to the preparation of standard curves by subject group of Guo et al. [30], the multiple mixed standard solution under “Preparation of Standard Solution” was injected into the instrument at the same time as the internal standard solution, and the determinations were performed sequentially; taking the average measurement value of 3 readings measured for each mass concentration as the ordinate (*y*) and the mass concentration of the standard solution corresponding elements as the abscissa (*x*), the standard curve was plotted; then linear regression was performed to obtain the regression equations of 18 metal elements, and the results are shown in Supplementary Table 2. The results showed that each element had a good linear relationship in the corresponding mass concentration range. The mixed standard solution was taken for six consecutive injections, the contents of 18 minerals and heavy metals were determined, and the RSD value of the response value of each element was calculated; see Supplementary Table 2 for details. The measurement precision of the 18 elements is good and meets the analysis requirements.

Referring to the repeatability experiment of Guo Hong Li's research group [30], and using the same way, 6 sample solutions were prepared in parallel, and the sample solutions and the internal standard solutions were injected into the instrument at the same time; then sequential determination was performed, and the RSD value of each element is shown in Supplementary Table 2. The RSD values of the 18 elements were all less than 5%, indicating good repeatability. In addition, accurately weighed 6 samples (QSW), each 0.0250g, referring to “Preparation of Test Samples of QSW” to prepare sample solutions and internal standard solutions were prepared according to “Preparation of Standard Solution,” then the sample solutions and internal standard solutions were injected into the instrument at the same time, and the recovery rates of 18 elements were calculated. The results are shown in Supplementary Table 2. The average sample recovery rate of 18 elements is 95.10%–111.13% (RSD, 2.13%–4.24%).

Referring to “Preparation of Test Samples of QSW,” the blank solution was prepared by the same method, injected into the instrument, and measured 11 times continuously, and the detection limit of each element was obtained. See Supplementary Table 2 for details.

#### 3.1.2. Content of Minerals and Heavy Metal Elements in QSW

After detection by ICP-MS, the contents of 18 elements in QSW are obtained: Li 2.893 *μ*g/kg, Be 1.504 *μ*g/kg, Sc 0.711 *μ*g/kg, V 7.025 *μ*g/kg, Cr 78.201 *μ*g/kg, Mn 1292.577 *μ*g/kg, Co 4.941 *μ*g/kg, Ni 35.643 *μ*g/kg, Cu 10240.028 *μ*g/kg, As 601.874 *μ*g/kg, Sr 242.575 *μ*g/kg, Ag 1025.272 *μ*g/kg, Cd 0.247 *μ*g/kg, Cs 0.387 *μ*g/kg, Ba 53.618 *μ*g/kg, Pb 10184.186 *μ*g/kg, Au 1098.911 *μ*g/kg, and Hg 5200.500 *μ*g/kg. The element content of QSW samples from high to low is Cu > Pb > Hg, which are all higher than 5000 *μ*g/kg; the contents of Mn, As, Ag, and Au are all higher than 600 *μ*g/kg; and the contents of Li, Be, Sc, V, Cr, Co, Ni, Sr, Cd, Cs, and Ba are all less than 300 *μ*g/kg.

#### 3.1.3. Content of Minerals and Heavy Metals in QSW after Digestion with Simulated GI Juice

The content of each element after GI digestion was determined. The contents of Mn, Cu, Sr, Pb, Au, and Hg in gastric juice (1 h) are significantly higher than those in water, which may be related to the easier dissolution of these trace elements under acidic conditions. The contents of Cu, Au, Sr, and As were higher in intestinal juice (4 h) than in water. In the simulated digestion experiment of artificial gastric juice and intestinal juice, the release changes of Cu and Pb are obvious: in gastric juice, the release of Cu and Pb was 17.65 and 30.56 *μ*g/kg, respectively; while in intestinal juice, the release of Cu and Pb was 4.406 and 0.764 *μ*g/kg, respectively, and the change of Pb is the most significant. Therefore, after the clinically used QSW are digested by artificial GI juice, the released heavy metal elements were very low and many elements have fallen below the detection limit. See Table 1 for details.

### 3.2. In Vivo Analysis of Minerals and Heavy Metals in QSW by ICP-MS

#### 3.2.1. In Vivo Processes of Minerals and Heavy Metals

As with previous experiments [20], the model was successful.  Table 2 shows that the absorption of Li into the blood is not obvious, which is mainly excreted through feces and urine, and almost no excretion through hair; there is a certain distribution in the brain, suggesting that it may have a certain relationship with the efficacy or toxicity of QSW. The absorption of Be into the blood is not obvious, and the Be absorbed into blood is almost not excreted through urine and hair, and feces are the main excretion route. The absorption of Sc into the blood is not obvious, and the Sc absorbed into the blood is almost not excreted through urine and hair, and feces are its main excretion route. V is obviously absorbed into the blood, which is mainly excreted through feces and urine but hardly through hair. Cr is absorbed into the blood obviously; the Cr absorbed into the blood is hardly excreted through urine and hair, mainly through feces; it is mainly distributed in the brain, suggesting that it may have a certain relationship with the efficacy or toxicity of QSW. The absorption of Mn into the blood is not obvious, and the Mn absorbed into the blood is almost not excreted through urine and hair but mainly through feces; it is mainly distributed in the liver. Co is absorbed into the blood obviously, the Co absorbed into the blood is hardly excreted through urine and hair, and feces are its main excretion route. Ni is absorbed into the blood obviously, the Ni absorbed into the blood is hardly excreted through urine and hair, and feces are its main excretion route. The absorption of Cu into the blood is not obvious, and the Cu absorbed into the blood is hardly excreted through urine and hair, and feces are its main excretion route. The absorption of As into the blood is not obvious, and the As absorbed into the blood is finally excreted through feces and urine, of which feces are the main route, and it is almost not excreted through hair. Sr is absorbed into the blood obviously, the more it is absorbed over time; the Sr absorbed into the blood is finally excreted through feces and urine, of which feces is the main route, and it is hardly excreted through hair. Ag is not obviously absorbed into the blood, mainly excreted through feces and urine but almost not through hair; there is a distribution in the kidney at 24 h; there is no obvious distribution in the brain. The absorption of Cd into the blood is not obvious, and it is mainly excreted through feces and urine, but almost not through hair; it is mainly distributed in the brain, suggesting that it may have a certain relationship with efficacy or toxicity of QSW. The absorption of Cs into the blood is not obvious, and it is mainly excreted through feces and urine, but almost not through hair; it is mainly distributed in the kidney. Ba is not obviously absorbed into the blood, and it is mainly excreted through feces, but almost not through urine and hair; it has no obvious distribution in the brain, liver, and kidney. The absorption of Pb into the blood is obvious, the Pb absorbed into the blood is finally excreted through feces and urine, of which feces are the main route, and it is almost not excreted through hair; it is mainly distributed in the liver and kidney. The absorption of Au into the blood is not obvious, and the Au absorbed into the blood is hardly excreted through urine and hair, and feces are its main excretion route. The absorption of Hg into the blood is not obvious, and the Hg absorbed into the blood is excreted through feces, urine, and hair, of which feces is the main path.

#### 3.2.2. Analysis of the Content of Minerals and Heavy Metals in Blood and Different Tissues

**Figure 1:**
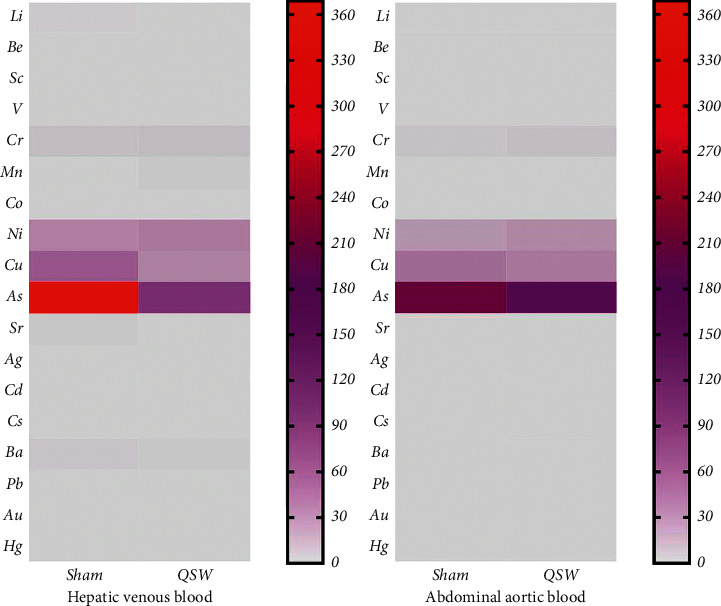
Heatmap of 18 elements in the artery and vein (*n* = 6). (*Note.* The redder the color, the higher the content; the grayer the color, the lower the content.)

Changes in the content of minerals and heavy metals in the blood are shown in Table 2 and Figure 1. In hepatic venous blood, the V content in the QSW + MCAO was significantly reduced compared with the Sham group (*p* < 0.05). In abdominal aortic blood, the contents of V, Cr, Co, Ni, and Sr were increased by QSW + MCAO (*p* < 0.05). However, as far as the content of each trace element in Table 2 is concerned, the trace element content between the Sham and QSW + MCAO groups is not different and the content is extremely low. Taking into account the trace elements in the blood itself, the differences between experimental animals, and the measurement sensitivity of ICP-MS itself, it can be considered that the content of trace elements and even heavy metals in the QSW + MCAO remained in the blood 24 h after successful modeling is extremely low.

**Figure 2:**
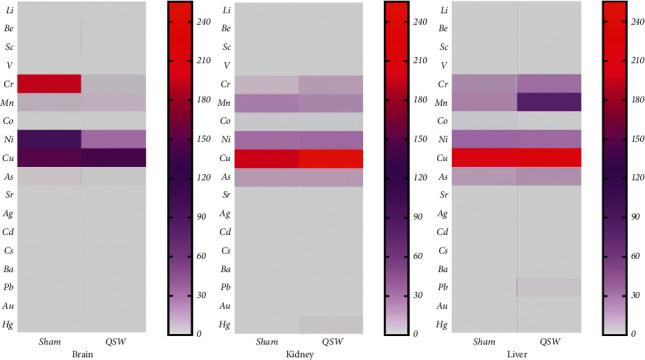
Heatmap of 18 elements in the brain, kidney, and liver (*n* = 6). (*Note.* The redder the color, the higher the content; the grayer the color, the lower the content.)

The brain, kidney, and liver were used to analyze the distribution and accumulation of minerals and heavy metals in the tissues, and the results are shown in Table 2 and Figure 2. In brain tissues, the content of Li increased by QSW + MCAO (*p* < 0.05), while the content of Cr and Cd decreased significantly (*p* < *0.05*). In kidney tissues, Ag and Cs contents increased by QSW + MCAO at 24 h of MCAO (*p* < 0.05). In liver tissues, the content of Mn increased by QSW + MCAO at 24 h of MCAO, while the content of Ni decreased (*p* < 0.05). The results showed that at 24 h of QSW + MCAO, the content of Mn in the liver was higher than that of the Sham group and was statistically significant (Table 2). Therefore, we speculate that the liver of QSW + MCAO rats is the site of Mn metabolism.

**Figure 3:**
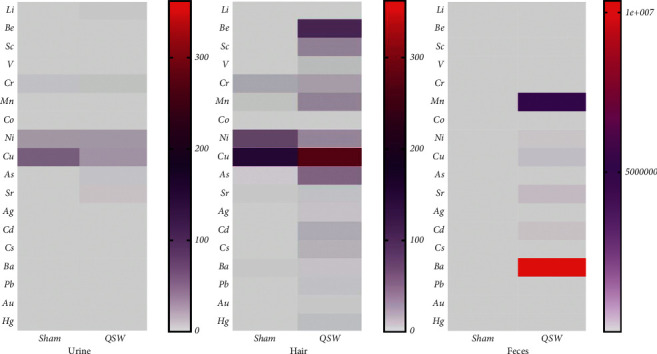
Heatmap of 18 elements in urine, hair, and feces (*n* = 6). (*Note.* The redder the color, the higher the content; the grayer the color, the lower the content.)

Urine, hair, and feces are main excretion routes for minerals, and the results are shown in Table 2 and Figure 3. In urine, at 24 h of QSW + MCAO, the contents of Li, V, Sr, Ag, Cd, Cs, and Hg increased significantly (*p* < 0.05). In the hair, the content of Ni decreased, while the content of Hg increased by QSW + MCAO (*p* < 0.05). In feces, at 24 h of MCAO, the contents of Li, V, Cr, Mn, Co, Cu, As, Sr, Ag, Cd, Cs, Ba, Pb, and Hg increased significantly (*p* < 0.05). Therefore, we speculate that the hair of QSW + MCAO rats could be the site of Hg metabolism. Although other elements are not statistically significant, their content is still increasing sharply (Be, Sc, Ni, Au). It can be seen that feces is the main excretion pathway of trace elements and heavy metals in QSW.

Compared with the content of minerals and heavy metals in QSW itself, it can be seen that the content of 18 elements in the abdominal aortic blood, hepatic venous blood, brain, liver, and kidney of QSW + MCAO rats is extremely low, the absorption is not high, and most of them are excreted through feces, and a few are excreted through urine and hair, which further proves taking QSW is relatively safe.

## 4. Discussion

This study concludes that the contents of Hg, Cu, Pb, Ag, As, Mn, and Au in QSW are relatively high. Under normal conditions, intake of such high levels of trace elements and even heavy metals is toxic to the human body when in excess amounts. However, after QSW were digested by artificial GI juice, the release of these heavy metals is very low, and many elements have fallen below the detection limit. These minerals and heavy metals must also be absorbed into the blood to exert their physiological effects in the human body. Hence, the low release of heavy metals may be acceptable. Finally, one QSW pill usually takes 3 to 7 days to be absorbed. At this time, the body's normal metabolism will also metabolize most of the trace elements. Therefore, the high content of trace elements and even heavy metals in QSW is not a sound basis to declare this Tibetan medicine as unsafe. The *Pharmacopoeia of the People's Republic of China* only conducts the microscopic identification of pearl, nine eye stone, and *Dalbergia odoratum* and the thin-layer identification of cholic acid, saffron, agarwood, benzoin, and safflower [4]. This process does not involve the quality control of trace elements or even heavy metals. However, heavy metals should have strict limits whether they are being used as toxic ingredients or as the basis of medicinal substances. At present, the research on minerals and heavy metals in QSW at home and abroad is still in infancy. No work has focused on the in vivo and in vitro aspects to systematically and quantitatively analyze the content of minerals and heavy metals in QSW. According to the in vitro simulated gastric digestion experiment in this work, the dissolution rate of other elements is higher than that of water extraction, except for the undetected and Ag elements. Two explanations for this phenomenon are presented. One is that hydrochloric acid destroys the internal structure of these minerals and heavy metals, thus allowing them to be dissolved in gastric juice. Another is the precipitation reaction of free silver dissolved in gastric juice and hydrochloric acid, thereby lowering the total silver content in gastric juice compared with that in water. In the simulated intestinal digestion experiment, the content of almost all detected minerals and heavy metals was far lower than that in the gastric juice.

Zuotai, an important component in QSW, is mainly made of mercury. It is not used alone in clinical practice but plays an important role in Tibetan medicine preparations, such as improving curative effects, reducing side effects, strengthening the spleen, and nourishing the body. [31]. Because Zuotai contains mercury, which is a toxic heavy metal element, its safety has received extensive attention. Previous studies [32] have conducted experiments on the dissolution of Zuotai in simulated GI, and the results showed that the dissolution of mercury in the simulated intestinal juice of Zuotai was significantly lower than that in the simulated gastric juice. Our study on QSW also showed the same result. Therefore, gastric juice digestion plays a key role in the absorption and metabolism of minerals and heavy metals in QSW.

The content of minerals and heavy metals in cerebral ischemia rats treated with QSW was determined. In our experiment, we also studied the QSW with a dose of 5 g/kg and executed them at 0.5 h after making the model. The results showed that there was no significant difference from the Sham group. The reason may be that the time is too short; the elements of QSW cannot be absorbed and distributed, even if its dose is large enough. These pilot results showed that it takes a certain period of time to produce effect after taking QSW.

When the rats were treated with QSW (66.68 mg/kg) first, then at 24 h of successful modeling, the Hg, Pb, Cu, As, and other heavy metal elements are mainly excreted in feces, and the heavy metals that are absorbed into the blood or accumulated in the brain, liver, kidney, and other tissues are extremely low. Nonetheless, in the measurement of elements accumulated in brain tissues, it was found that the content of Li increased, and the content of Cd and Cr decreased, and both were significant; Li was absorbed into the body and mainly distributed in the brain, indicating that its main target site to exert its pharmacological or toxic effects is the brain. Hence, Li is possibly to be the material basis of QSW for the treatment of cerebral ischemia. According to reports in the literature, Li can improve the integrity of the blood-brain barrier, sensorimotor deficits, and cerebral edema in animals with cerebral hemorrhage [33]. The excretion of minerals in the feces is the main route of disposition of these elements, including the above three. Considering their long-term stay in the GI tract, the gut-microbiota-brain axis in QSW-mediated brain protection is evident [21].

QSW is rich in minerals and heavy metals than other Chinese patent medicines. These toxic elements cause strong toxic reactions only when in their free form and in excess; if minerals and heavy metals are combined with other macromolecular compounds, then their toxic reactions are greatly weakened or directly converted into nontoxic chemical compounds [34]. Heavy metals are generally viewed as toxic to the human body. From the perspective of traditional medicine, toxicity can be reduced by processing or strict dosage control; “both poison and medicine” is a relevant saying. Main ingredients, such as Zuotai of QSW, must undergo “mercury refining method” [35] to ensure that the toxicity of Hg and other metal elements is greatly reduced or even removed. When metals enter the body, complex physiological changes may cause them to combine with certain macromolecular substances to reduce their toxicity or exert pharmacological effects. For example, when Cd and Cr are absorbed into the blood and are distributed in the brain, they may combine with some macromolecular substances to exhibit a therapeutic effect. Whether decreased Cd and Cr in the brain by QSW could contribute to its neuroprotection is unknown and worth investigating. QSW increased Ag and Cs in the kidney and increased Mn but decreased Ni in the liver; the biological significance of such changes also requires further investigation. In addition, the human body's absorption of mercury and other minerals is low mainly because most of them are excreted in feces, which indicates the importance of the gut-microbiota-brain axis in QSW-mediated brain protection; secondly, the content of trace elements of the human body required is not high, although the absorption is low, it may just meet the needs of the human body. Moreover, the Li, Cr, Cd, and other elements detected in brain tissues are likely to be the material basis for QSW to treat cerebral ischemia.

However, what about the accumulation of QSW in the body after long-term use? The dosage of QSW in the instructions is 1 g (1 pill) per day for critically ill patients, and general patients take 1 g every 3 to7 days. In this study, rats were given the clinically equivalent dose of QSW, that is, the optimal dose (66.68 mg/kg), and within 24 h after the successful modeling, most of the minerals and heavy metals have been excreted through urine or feces, and then after 3 to 7 days, the minerals and heavy metals in the body may be less. Therefore, whether it is for critically ill patients or general patients, the accumulation of minerals and heavy metals in the body after long-term use of QSW is very small and basically does not accumulate; it is safe to take QSW.

In our experiment, the results showed that these elements can be detected in the Sham group. These trace elements could be essential components in the body. This study is the first to use ICP-MS to examine the in vivo absorption, distribution, and excretion of minerals and heavy metals from QSW and further objectively prove its safety. However, there is also one deficiency, that is, we did not set up the model control group (MCAO group), and we do not know whether it will affect the content of these elements in the body after modeling. Nonetheless, Sham rats without receiving QSW provided a basal control for absorption, distribution, and excretion of elements following QSW administration.

## 5. Conclusions

Quantifying the minerals and heavy metals in QSW in vivo and in vitro will help improve its quality standards. The abundant minerals and heavy metals in this medicine may either be the basis of its main medicinal substances or promote the therapeutic effects of its effective ingredients. The absorption, distribution, and excretion of minerals and heavy metals from QSW in MCAO rats add to our understanding of neuroprotective effects of QSW. The accumulation of the elements in blood and tissues was extremely low, and the majority was excreted in feces within 24 h, highlighting the importance of the gut-microbiota-brain axis in QSW-mediated brain protection.

## Figures and Tables

**Table 1:** Determination results of contents of 18 elements in GI juice (*μ*g/kg).

Element	Water extraction (1 h)/(*μ*g/kg)	Stomach digestion (1 h)/(*μ*g/kg)	Water extraction (4 h)/(*μ*g/kg)	Intestinal digestion (4 h)/(*μ*g/kg)
**Li**	**0.044**	—	0.094	0.049
**Be**	—	—	—	—
**Sc**	—	—	—	—
**V**	—	—	—	—
**Cr**	—	—	—	—
**Mn**	0.518	6.179	—	—
**Co**	0.040	0.081	0.011	0.019
**Ni**	2.653	—	—	—
**Cu**	2.292	17.651	0.853	4.406
**As**	3.663	4.228	0.581	0.845
**Sr**	0.446	8.598	0.080	0.928
**Ag**	72.985	51.974	45.854	41.845
**Cd**	—	—	—	—
**Cs**	0.004	0.013	—	0.006
**Ba**	—	—	—	—
**Pb**	1.686	30.558	1.269	0.764
**Au**	1.790	4.846	0.903	2.959
**Hg**	1.170	4.518	3.406	1.102

The symbol “—” means not detected or lower than the detection limit. Bold indicates that the data are consistent with other data.

**Table 2:** Changes in the contents of 18 elements in rats with cerebral ischemia (*n* = 6, *μ*g/kg).

Element	Group	Absorption		Distribution			Excretion		
		Hepatic venous blood	Abdominal aortic blood	Brain	Kidney	Liver	Urine	Hair	Feces
Li	Sham	1.799 ± 1.469	0.573 ± 0.240	0.021 ± 0.027	0.025 ± 0.035	—	0.298 ± 0.082	0.603 ± 0.128	0.9705 ± 0.03541
	QSW + MCAO	0.846 ± 0.119	0.827 ± 0.227	0.121 ± 0.053^*∗*^	0.261 ± 0.148	0.112 ± 0.113	2.921 ± 1.687^*∗*^	1.658 ± 0.417	22.74 ± 2.601^*∗*^

Be	Sham	0.041 ± 0.070	0.007 ± 0.008	0.021 ± 0.028	0.006 ± 0.008	0.006 ± 0.008	0.006 ± 0.007	0.002 ± 0.031	0.0447 ± 0.0216
	QSW + MCAO	0.015 ± 0.018	0.002 ± 0.006	—	0.002 ± 0.004	0.026 ± 0.058	0.005 ± 0.007	105.5 ± 211.1	87.93 ± 0.660

Sc	Sham	0.018 ± 0.024	0.002 ± 0.002	0.016 ± 0.013	0.002 ± 0.002	0.021 ± 0.028	0.001 ± 0.002	0.019 ± 0.007	0.0434 ± 0.0171
	QSW + MCAO	0.001 ± 0.001	0.001 ± 0.001	0.006 ± 0.005	0.008 ± 0.006	0.012 ± 0.007	0.004 ± 0.004	40.63 ± 81.18	95.76 ± 14.48

V	Sham	0.430 ± 0.328	0.029 ± 0.005	0.441 ± 0.072	0.736 ± 0.486	0.845 ± 0.664	0.023 ± 0.004	0.319 ± 0.033	11.28 ± 0.532
	QSW + MCAO	0.070 ± 0.087^*∗*^	0.045 ± 0.011^*∗*^	0.132 ± 0.015	1.057 ± 0.341	0.524 ± 0.199	0.068 ± 0.033^*∗*^	11.16 ± 21.32	35.49 ± 23.53^*∗*^

Cr	Sham	9.508 ± 6.316	7.412 ± 1.188	192.2 ± 26.37	9.153 ± 0.409	25.71 ± 18.07	6.913 ± 0.679	21.61 ± 1.308	0.912 ± 0.6191
	QSW + MCAO	11.09 ± 0.701	9.344 ± 0.713^*∗*^	9.889 ± 1.242^*∗*^	17.99 ± 16.83	36.01 ± 19.77	7.268 ± 0.506	25.31 ± 21.61	12.740 ± 8.081^*∗*^

Mn	Sham	1.166 ± 1.643	0.027 ± 0.055	12.38 ± 3.375	30.41 ± 12.66	29.13 ± 0.699	0.038 ± 0.075	7.336 ± 0.793	330.8 ± 167.5
	QSW + MCAO	2.246 ± 5.481	0.009 ± 0.022	10.08 ± 1.656	262.3 ± 52.30	80.75 ± 10.24^*∗*^	0.241 ± 0.248	40.16 ± 30.79	50480 ± 27940^*∗*^

Co	Sham	0.044 ± 0.023	0.013 ± 0.006	0.493 ± 0.272	1.481 ± 1.650	1.395 ± 1.362	0.135 ± 0.177	0.115 ± 0.016	0.2862 ± 0.1663
	QSW + MCAO	0.047 ± 0.053	0.026 ± 0.004^*∗*^	0.089 ± 0.011	1.783 ± 0.303	0.589 ± 0.116	0.139 ± 0.074	0.656 ± 0.748	42.86 ± 9.576^*∗*^

Ni	Sham	41.84 ± 21.08	31.91 ± 4.188	977.7 ± 616.2	36.22 ± 0.577	38.97 ± 0.064	28.98 ± 2.942	77.52 ± 12.03	30.60 ± 13.27
	QSW + MCAO	46.04 ± 2.302	37.96 ± 3.100^*∗*^	37.06 ± 4.362	36.94 ± 1.428	36.74 ± 1.222^*∗*^	28.79 ± 2.422	38.78 ± 0.989^*∗*^	1460 ± 1977

Cu	Sham	66.99 ± 47.98	54.53 ± 48.46	147.2 ± 17.60	196.4 ± 2.363	206.3 ± 24.72	59.11 ± 39.59	147.6 ± 5.267	12.68 ± 62.45
	QSW + MCAO	41.11 ± 41.22	46.93 ± 44.93	140.3 ± 9.885	255.4 ± 48.35	220.1 ± 13.79	30.79 ± 44.10	269.9 ± 105.1	2325 ± 191.3^*∗*^

As	Sham	368.9 ± 269.3	209.02 ± 74.28	3.215 ± 1.613	19.27 ± 6.584	19.73 ± 4.745	1.541 ± 0.038	1.792 ± 0.352	0.2964 ± 0.1596
	QSW + MCAO	101.2 ± 3.592	157.4 ± 25.59	2.042 ± 0.275	19.59 ± 4.862	23.95 ± 5.165	4.224 ± 0.464	55.86 ± 104.4	81.92 ± 9.358^*∗*^

Sr	Sham	3.028 ± 4.483	0.026 ± 0.052	0.658 ± 0.299	0.262 ± 0.371	0.598 ± 0.042	0.581 ± 0.082	2.085 ± 0.1211	18.63 ± 7.826
	QSW + MCAO	1.156 ± 2.427	0.451 ± 0.132^*∗*^	0.516 ± 0.144	1.055 ± 0.492	0.265 ± 0.494	5.612 ± 3.481^*∗*^	6.876 ± 5.843	3091 ± 1423^*∗*^

Ag	Sham	0.116 ± 0.053	0.011 ± 0.014	0.854 ± 1.242	0.016 ± 0.001	0.091 ± 0.079	—	0.632 ± 0.979	0.1119 ± 0.01198
	QSW + MCAO	0.130 ± 0.150	0.020 ± 0.023	0.142 ± 0.079	0.195 ± 0.097^*∗*^	0.147 ± 0.091	0.058 ± 0.036	5.781 ± 10.01	151.30 ± 69.56^*∗*^

Cd	Sham	0.030 ± 0.034	—	0.064 ± 0.031	0.229 ± 0.086	0.232 ± 0.095	—	0.025 ± 0.008	0.0107 ± 0.0559
	QSW + MCAO	0.022 ± 0.038	0.001 ± 0.002	0.003 ± 0.004^*∗*^	0.153 ± 0.088	0.152 ± 0.076	0.005 ± 0.004	18.79 ± 37.49	2063 ± 203.9^*∗*^

Cs	Sham	0.119 ± 0.076	0.051 ± 0.019	0.269 ± 0.047	0.435 ± 0.007	0.509 ± 0.105	0.069 ± 0.001	0.056 ± 0.005	0.0156 ± 0.0056
	QSW + MCAO	0.054 ± 0.010	0.078 ± 0.009	0.296 ± 0.068	0.908 ± 0.167^*∗*^	0.782 ± 0.097	0.233 ± 0.114^*∗*^	15.02 ± 29.69	242.5 ± 113.5^*∗*^

Ba	Sham	3.597 ± 3.848	1.477 ± 1.124	0.512 ± 0.465	0.235 ± 0.332	0.453 ± 0.634	—	2.103 ± 0.291	4.731 ± 1.736
	QSW + MCAO	2.816 ± 5.429	0.352 ± 0.233	0.580 ± 1.037	0.662 ± 0.559	0.257 ± 0.500	—	6.439 ± 6.517	10360 ± 8227^*∗*^

Pb	Sham	0.925 ± 1.059	—	—	—	0.161 ± 0.028	—	0.830 ± 0.362	0.2564 ± 0.1205
	QSW + MCAO	0.477 ± 1.168	—	0.122 ± 0.176	1.216 ± 0.951	2.672 ± 2.554	0.204 ± 0.378	6.747 ± 3.947	198.40 ± 154.50^*∗*^

Au	Sham	0.024 ± 0.027	—	0.003 ± 0.006	0.013 ± 0.018	0.007 ± 0.009	—	—	0.0131 ± 0.0204
	QSW + MCAO	0.010 ± 0.017	0.004 ± 0.006	0.020 ± 0.029	0.351 ± 0.372	0.179 ± 0.207	0.022 ± 0.043	3.171 ± 3.106	12.01 ± 23.46

Hg	Sham	0.630 ± 0.394	0.429 ± 0.174	0.283 ± 0.099	0.468 ± 0.423	0.592 ± 0.445	0.050 ± 0.012	0.624 ± 0.103	0.0117 ± 0.005
	QSW + MCAO	0.460 ± 0.226	0.359 ± 0.032	0.358 ± 0.112	2.998 ± 1.231	0.606 ± 0.083	0.794 ± 0.446^*∗*^	9.332 ± 2.793^*∗*^	118.50 ± 84.31^*∗*^

The symbol “—” means not detected or lower than the detection limit; compared with the Sham group, ^*∗*^*p* < 0.05.

## Data Availability

Data supporting the conclusions are included in the manuscript. The data sets used and/or analyzed during the current study are also available from the corresponding author on reasonable request.

## Abbreviations

QSW: Qishiwei Zhenzhu pills
ICP-MS: Inductively coupled plasma mass spectrometry
GI: Gastrointestinal
MCAO: Middle cerebral artery occlusion
ICP-AES: Inductively coupled plasma-atomic emission spectrometry
SR-XRF: Synchrotron radiation X-ray fluorescence
FAAS: Flame atomic absorption
GAE-AFS: Gold amalgam enrichment-atomic fluorescence
ISS: Internal standard stock solution
SS: Standard solution
PTEF: Polytetrafluoroethylene
CCA: Common carotid artery
ECA: External carotid artery
ICA: Internal carotid artery.

## Supplementary Materials

Supplementary Materials
*Supplementary Table 1.* Composition of QSW. *Supplementary Table 2*. Results of linear equations and correlation coefficients of 18 elements.
