# Supplementary material for: The Absorption, Distribution, and Excretion of 18 Elements of Tibetan Medicine Qishiwei Zhenzhu Pills in Rats with Cerebral Ischemia
Source: Evid Based Complement Alternat Med. 2021 Dec 28;2021:4508533. doi: 10.1155/2021/4508533 (PMC8727113; doi:10.1155/2021/4508533)
Supplement: Supplementary Materials — Supplementary Table 1. Composition of QSW. Supplementary Table 2. Results of linear equations and correlation coefficients of 18 elements. [file 4508533.f1.docx]

Supplementary Table

**Supplementary** **Table 1 Composition of QSW**

| **Medicinal material types** | **Latin name** | **English name** | **Pinyin name (Chinese name)** |
| --- | --- | --- | --- |
| **Plant medicine** | *Crocus sativus* L. | Saffron | Xihonghua |
|  | *Santalum album* L. | Sandalwood | Tanxiang |
|  | *Benzoinum* | Benzoin | Anxixiang |
|  | *Aquilaria agallocha(Lour.)* Roxb | Agilawood | Chenxiang |
|  | *Dalbergia odorifera* | —— | Jiangxiang |
|  | *Lagotis brachystachya* Maxim. | —— | Duansuituercao |
|  | *Glycyrrhiza uralensis* Fisch. | Licorice | Gancao |
|  | *Bambusae concretio silicea* | —— | Tianzhuhuang |
|  | —— | —— | Muhezi |
|  | *Terminalia chebula* Retz. | Myrobalan | Hezi |
|  | *Nepeta angustifolia* C. Y. Wu | Tibetan fineleaf  schizonepeta herb | Zangjingjie |
|  | *Nepeta cataria* L. | Herba schizonepetae | Jingjie |
| **Animal medicine** | *Moschus* | Musk | Shexiang |
|  | —— | Buffalo blood | Yeniuxue |
|  | *Bos taurus domesticus* Gmelin | Bezoar | Niuhuang |
|  | *Saigae tataricae cornu* | Antelope horn | Lingyangjiao |
|  | —— | Bear bile | Xiongdan |
| **Mineral and gemstone medicine** | —— | Pearl | Zhenzhu |
|  | —— | Nine's eye | Jiuyanshi |
|  | —— | Agate | Manao |
|  | —— | Coral | Shanhu |
|  | —— | Sapphire | Lanbaoshi |
|  | —— | Cat's eye | Maoyanshi |
|  | —— | Tophus | Songshi |
|  | —— | Lapis lazuli | Qingjinshi |
| **Metal medicine** | —— | Gold (Au) | Jin |
|  | —— | Silver (Ag) | Yin |
|  | —— | Copper (Cu) | Tong |
|  | —— | Iron (Fe) | Tie |
| **Mixture** | —— | —— | Zuotai |

“——” means no Latin name or English name not found or no.

**Supplementary** **Table 2 Results of linear equations and correlation coefficients of 18 elements**

| **Element** | **Regression equation** | **R^2^** | **BEC**  **（ng/mL）** | **Linear range（μg/L）** | **The detection limit（ng/mL）** | **Repeatability**  **RSD/%** | **Precision**  **RSD/%** | **The average recovery rate** | **Sample recovery rate RSD/%** |
| --- | --- | --- | --- | --- | --- | --- | --- | --- | --- |
| **Li** | Y=183.4523X+30.0102 | 0.9997 | 0.164 | 0~50 | 0.0111 | 3.24 | 3.92 | 95.97 | 3.77 |
| **Be** | Y=120.4196X+1.6462 | 0.9997 | 0.014 | 0~50 | 0.0710 | 3.45 | 4.40 | 100.82 | 4.24 |
| **Sc** | Y=3568.7244X+38.2374 | 0.9999 | 0.011 | 0~50 | 0.0058 | 3.64 | 2.03 | 105.03 | 2.57 |
| **V** | Y=11335.3413X+785.6943 | 0.9999 | 0.069 | 0~50 | 0.0186 | 1.43 | 2.72 | 102.21 | 4.17 |
| **Cr** | Y=18508.5550X+9806.2069 | 0.9998 | 0.530 | 0~50000 | 0.0445 | 4.33 | 1.56 | 99.50 | 3.40 |
| **Mn** | Y=8186.2987X+5839.4773 | 0.9999 | 0.713 | 0~50000 | 0.0631 | 4.44 | 1.26 | 99.60 | 3.60 |
| **Co** | Y=33478.7095X+423.5628 | 0.9998 | 0.013 | 0~50 | 0.0030 | 4.05 | 2.35 | 96.56 | 3.81 |
| **Ni** | Y=8849.3395X+2884.8174 | 0.9999 | 0.326 | 0~50000 | 0.0611 | 1.66 | 2.52 | 104.69 | 2.13 |
| **Cu** | Y=23141.6409X+27031.1966 | 0.9992 | 1.168 | 0~50000 | 0.0769 | 4.32 | 1.39 | 99.31 | 3.73 |
| **As** | Y=1441.6875X+193.1546 | 0.9999 | 0.134 | 0~50000 | 0.0709 | 1.97 | 1.54 | 106.64 | 3.03 |
| **Sr** | Y=10750.8836X+18797.6326 | 0.9990 | 1.748 | 0~50000 | 0.0825 | 4.19 | 2.52 | 111.09 | 2.95 |
| **Ag** | Y=56823.9278X+172271.4074 | 0.9882 | 3.032 | 0~50000 | 0.2512 | 1.60 | 1.68 | 111.13 | 3.58 |
| **Cd** | Y=8600.8383X+114.9958 | 0.9998 | 0.013 | 0~50 | 0.0108 | 3.65 | 3.27 | 98.93 | 4.13 |
| **Cs** | Y=42739.5454X+791.7825 | 0.9995 | 0.019 | 0~50 | 0.0026 | 3.09 | 2.58 | 97.47 | 3.92 |
| **Ba** | Y=8843.5772X+38308.4026 | 0.9993 | 4.332 | 0~50000 | 0.1084 | 4.10 | 2.03 | 95.10 | 3.43 |
| **Pb** | Y=169100.0638X+223195.9483 | 0.9996 | 1.320 | 0~50000 | 0.1003 | 4.33 | 4.18 | 96.45 | 3.39 |
| **Au** | Y=66686.2742X+38970.8818 | 0.9999 | 0.584 | 0~50000 | 0.0517 | 4.31 | 3.82 | 99.96 | 3.22 |
| **Hg** | Y=20700.5877X+1037.7679 | 0.9989 | 0.050 | 0~200 | 0.0101 | 2.72 | 1.95 | 101.97 | 3.86 |
